# Supplementary material for: The anatomy and phylogenetic position of the erythrosuchid archosauriform Guchengosuchus shiguaiensis from the earliest Middle Triassic of China
Source: PeerJ. 2019 Feb 19;7:e6435. doi: 10.7717/peerj.6435 (PMC6385703; doi:10.7717/peerj.6435)
Supplement: Supplemental Information 1 — Additional figures of the type material of Guchengosuchus, forming part of the raw data underpinning the manuscript. [file peerj-07-6435-s001.pdf]

SUPPLEMENT TO:

The anatomy and phylogenetic position of the erythrosuchid archosauriform *Guchengosuchus shiguaiensis* from the earliest Middle Triassic of China

Richard J. Butler[1], Martín D. Ezcurra[1,2], Jun Liu[3,4], Roland B. Sookias[5], and Corwin Sullivan[3,6,7]

[1]School of Geography, Earth and Environmental Sciences, University of Birmingham, Edgbaston, Birmingham B15 2TT, UK

[2]Sección Paleontología de Vertebrados, CONICET–Museo Argentino de Ciencias Naturales "Bernardino Rivadavia", Av. Ángel Gallardo 470, C1405DJR, Buenos Aires, Argentina

[3]Key Laboratory of Vertebrate Evolution and Human Origins of Chinese Academy of Sciences, Institute of Vertebrate Paleontology and Paleoanthropology, Chinese Academy of Sciences Beijing 100044, China

[4]CAS Center for Excellence in Life and Paleoenvironment, Beijing, 100044, China

[5]Museum für Naturkunde, Leibniz Institute for Evolution and Biodiversity Science, Invalidenstraße 43, 10115 Berlin, Germany

[6]Department of Biological Sciences, University of Alberta, Edmonton, Alberta, T6G 2E9, Canada

[7]Philip J. Currie Dinosaur Museum, Wembley, Alberta, T0H 3S0, Canada

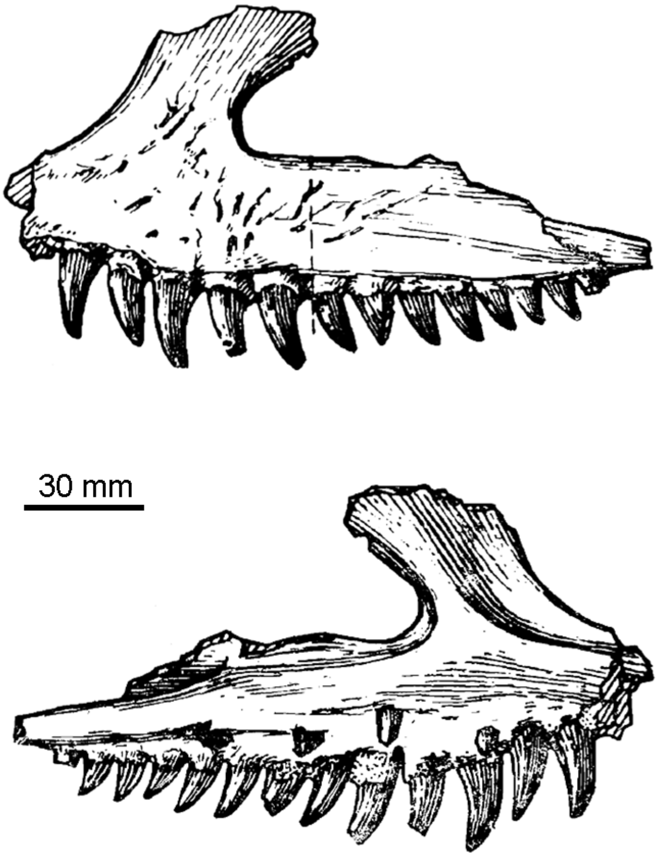

Figure S1. Maxilla of *Guchengosuchus shiguaiensis*, IVPP V8808, as originally preserved and figured by Peng (1991). Line drawings show the left maxilla in lateral (top) and medial (bottom) views.

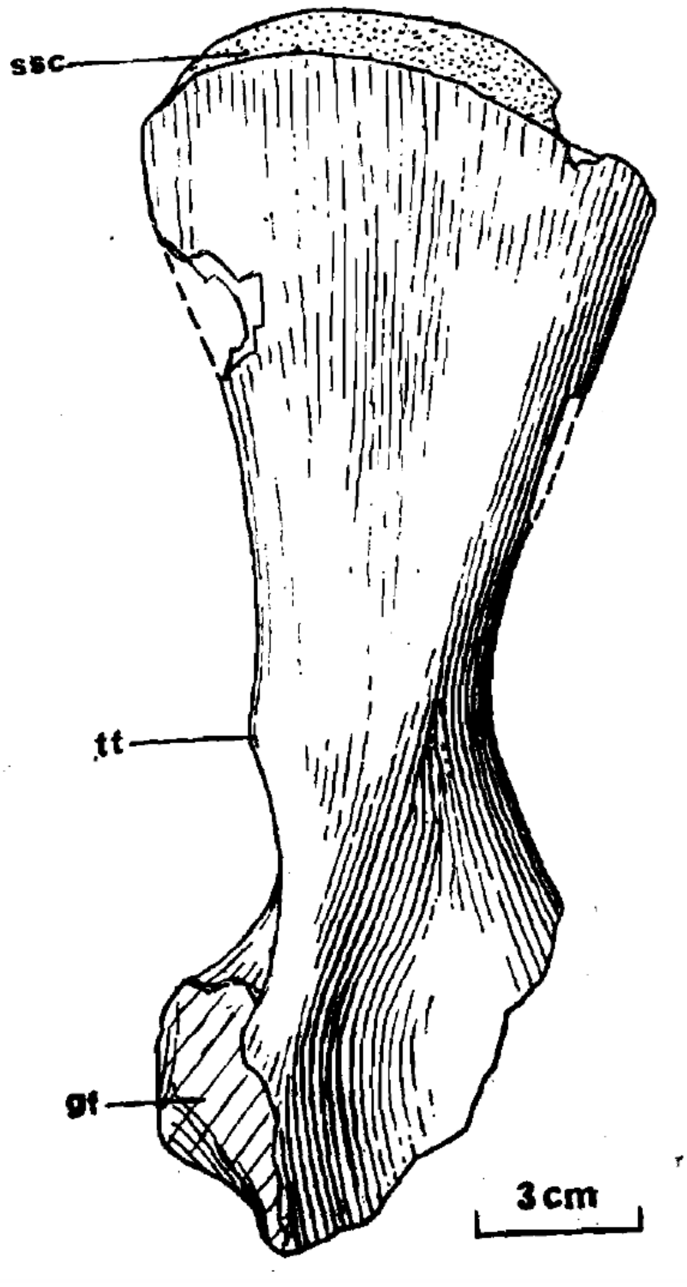

Figure S2. Right scapula of *Guchengosuchus shiguaiensis*, IVPP V8808, in lateral view, as originally preserved and figured by Peng (1991).

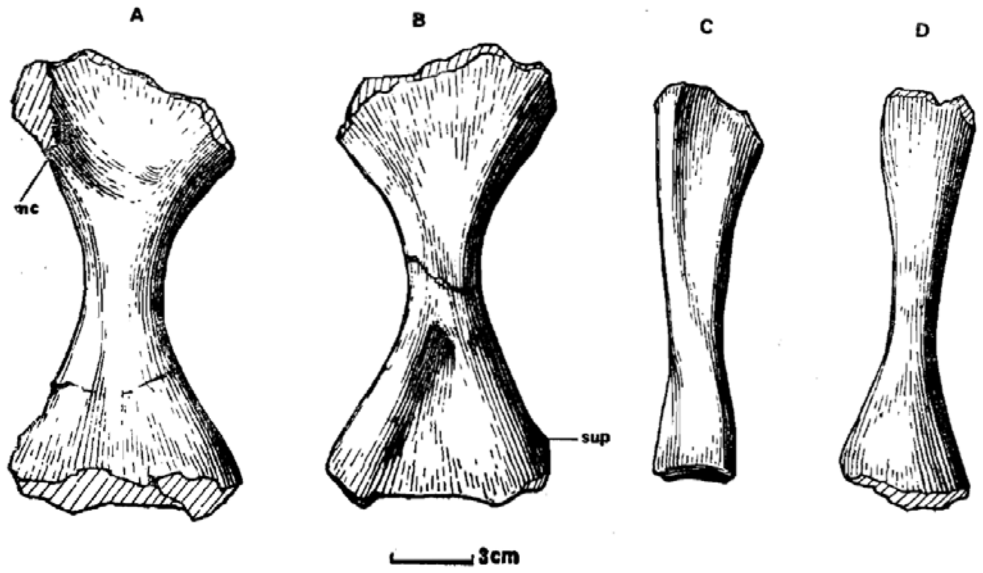

Figure S3. Postcranial elements of *Guchengosuchus shiguaiensis*, IVPP V8808, as originally preserved and figured by Peng (1991). Right humerus in anterior (A) and posterior (B) views. Right ulna (C) and right radius (D).

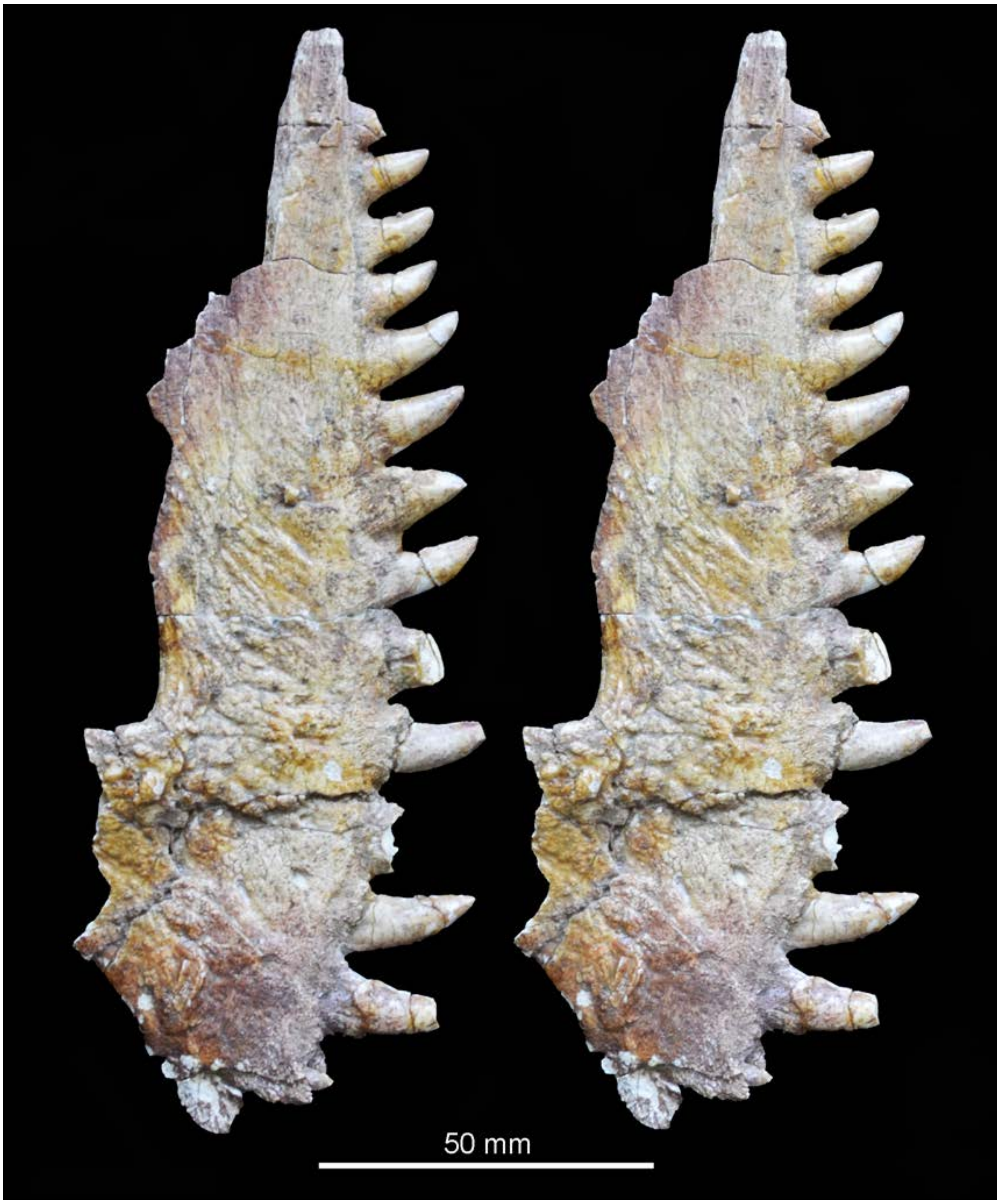

Figure S4. Left maxilla of *Guchengosuchus shiguaiensis*, IVPP V8808, stereopairs in lateral view.

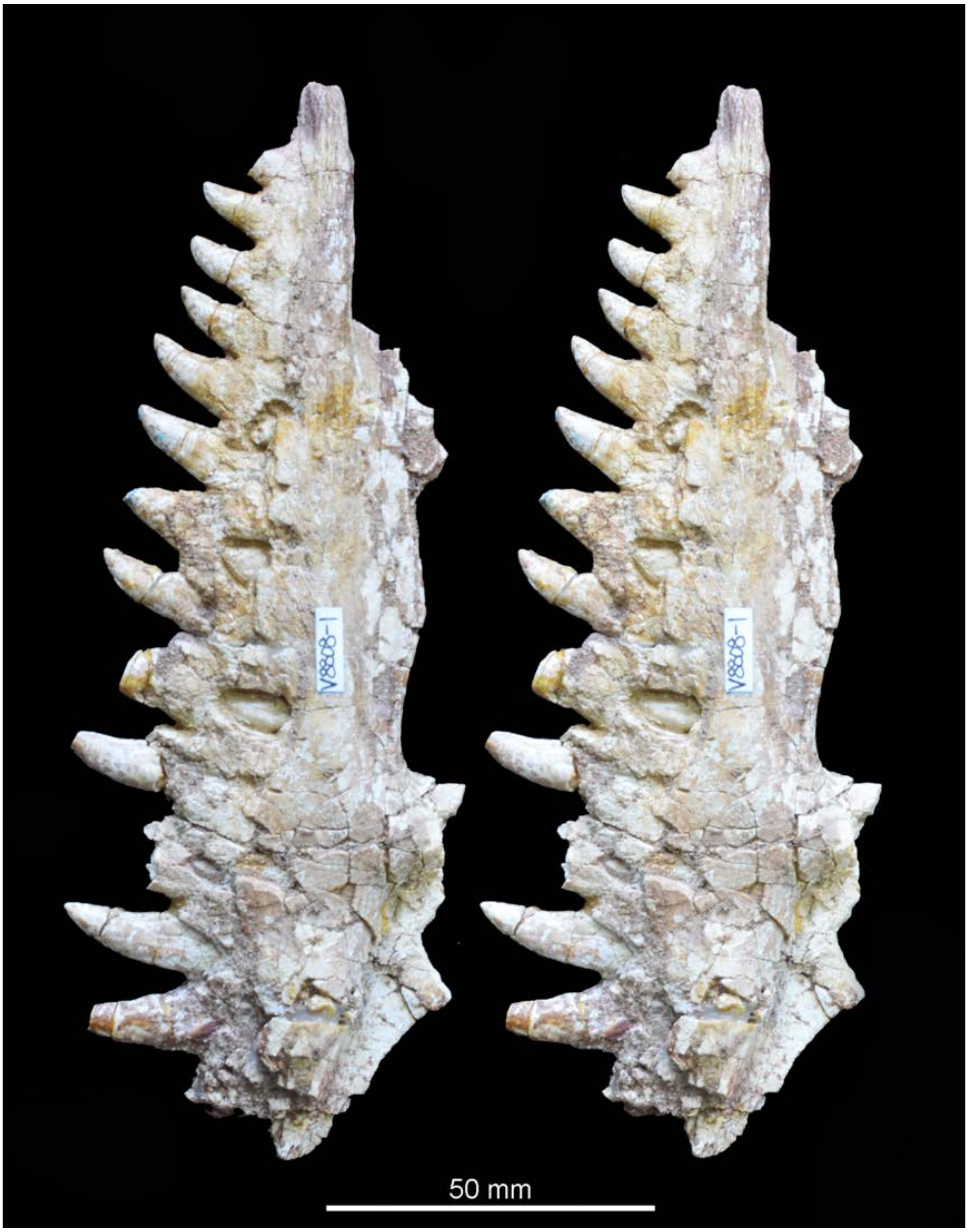

Figure S5. Left maxilla of *Guchengosuchus shiguaiensis*, IVPP V8808, stereopairs in medial view.

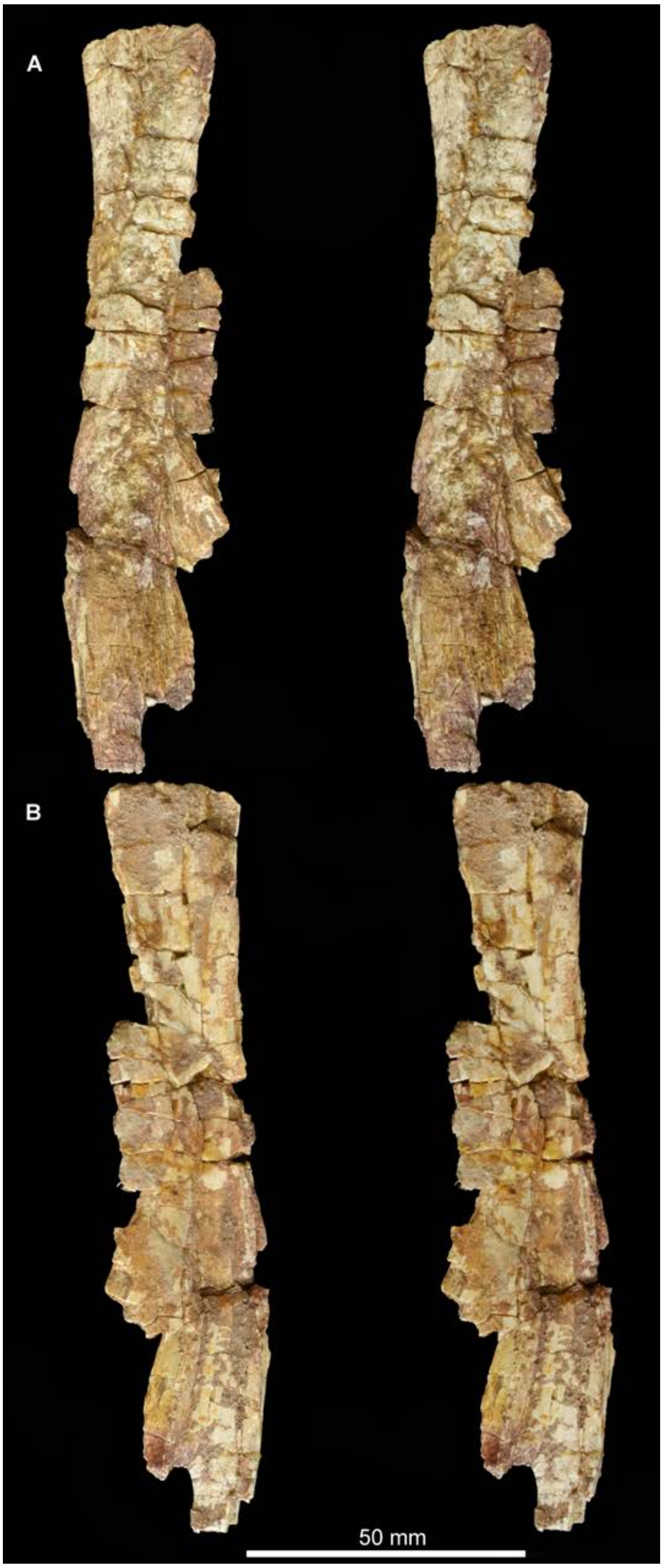

Figure S6. Left nasal of *Guchengosuchus shiguaiensis*, IVPP V8808, stereopairs in lateral (A) and medial (B) views.

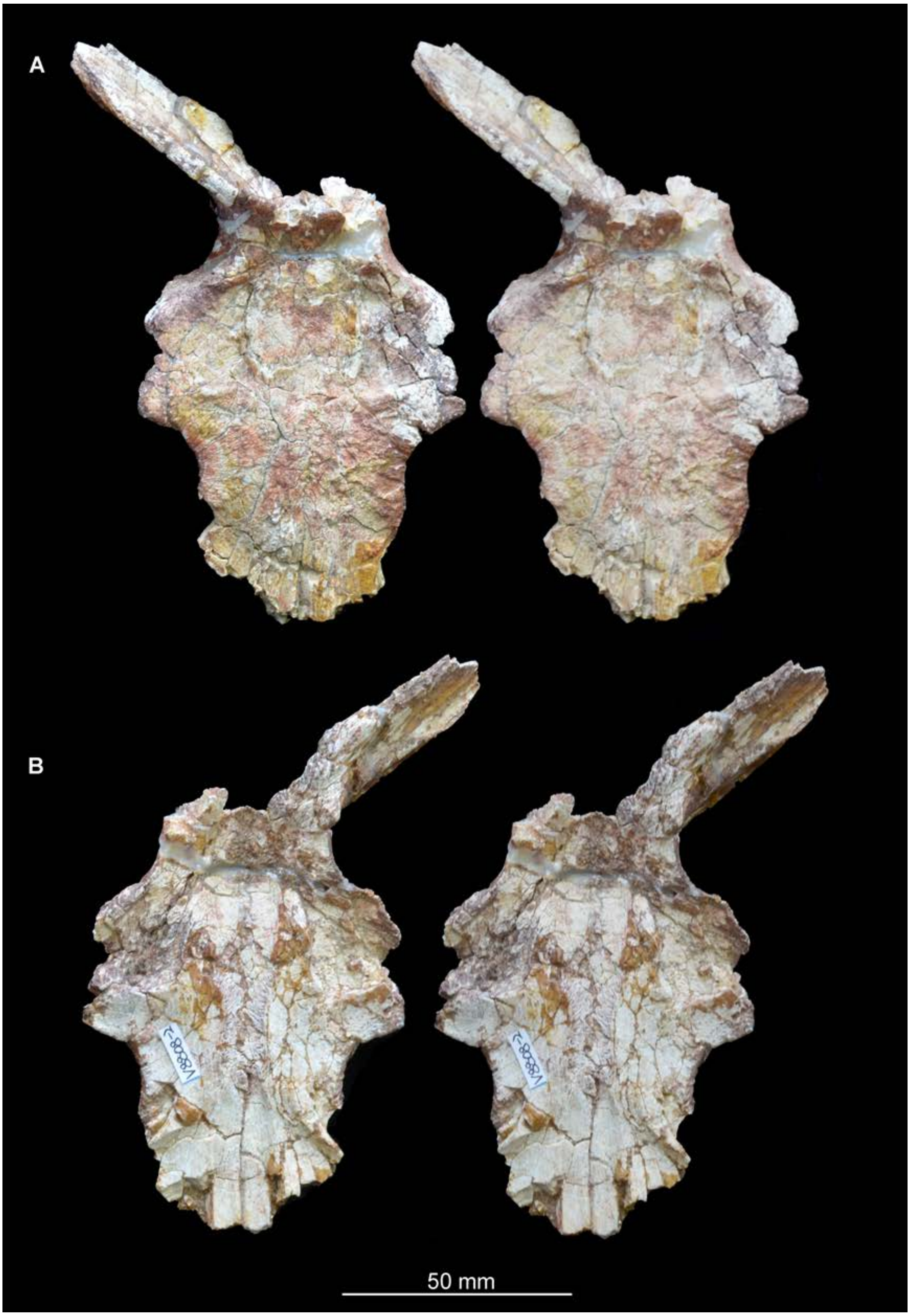

Figure S7. Skull roof of *Guchengosuchus shiguaiensis*, IVPP V8808, stereopairs in dorsal (A) and ventral (B) views.

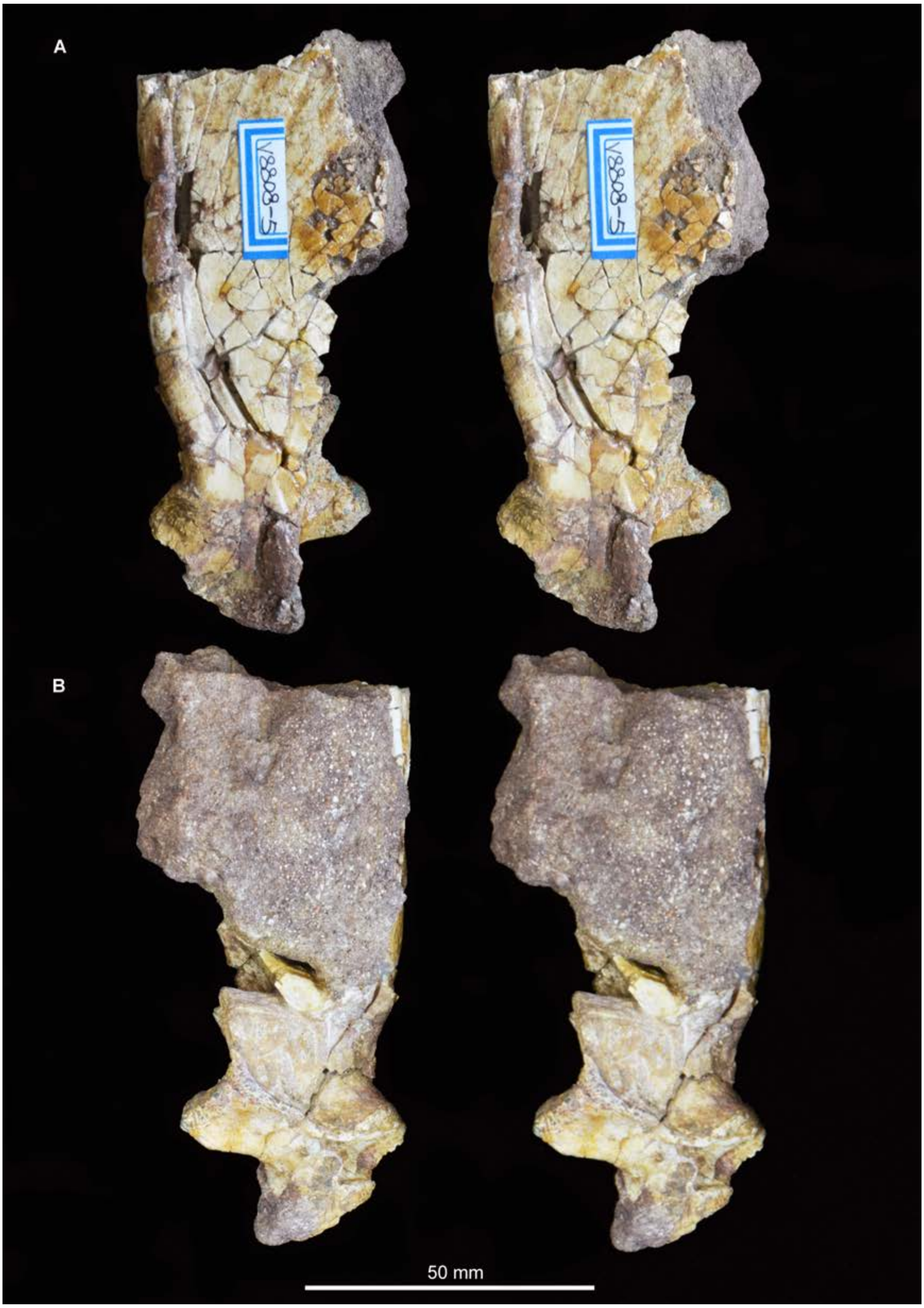

Figure S8. Posterior right mandible of *Guchengosuchus shiguaiensis*, IVPP V8808, stereopairs in lateral (A) and medial (B) views.

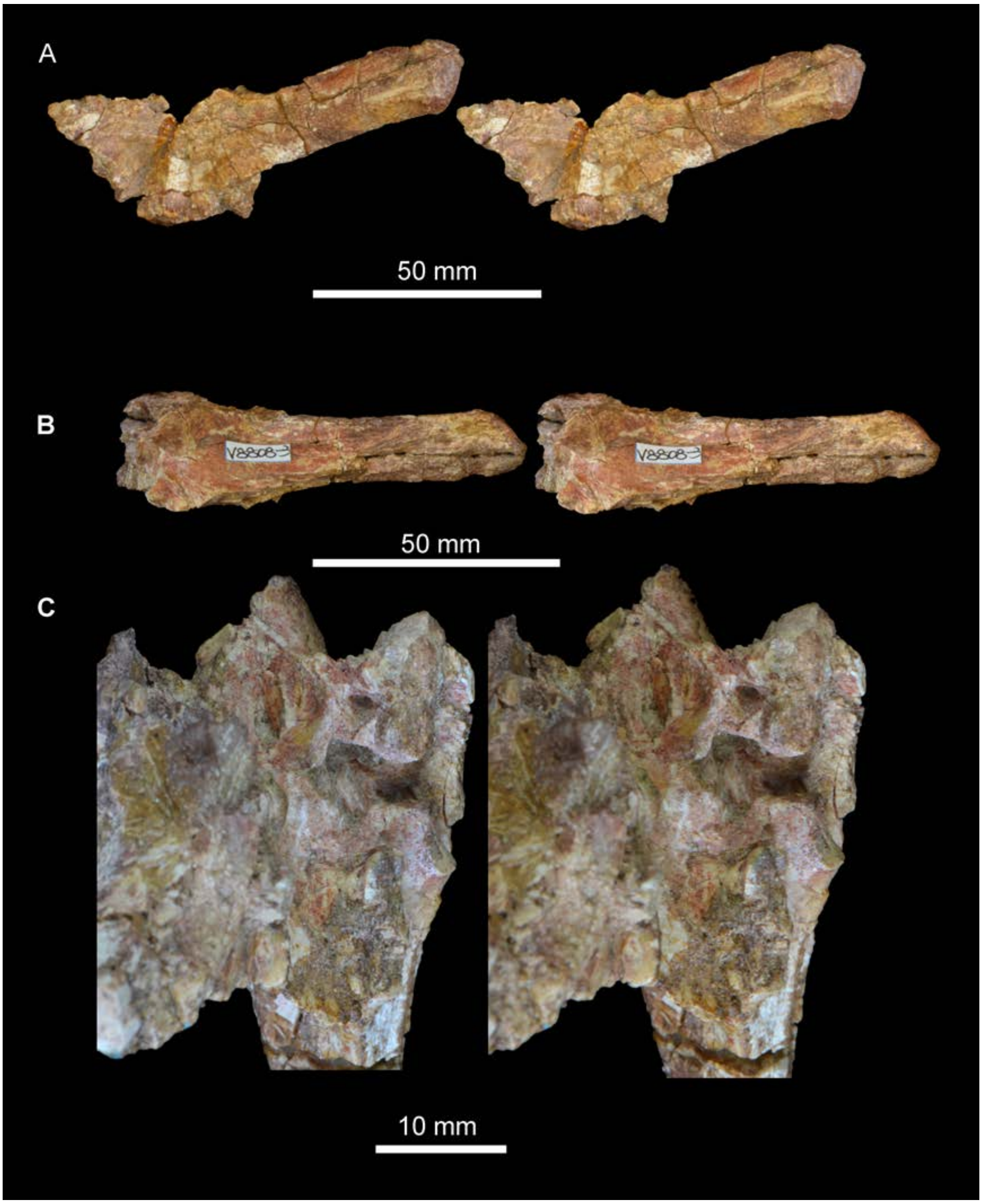

Figure S9. Braincase elements of *Guchengosuchus shiguaiensis*, IVPP V8808, stereopairs. Posterior view (A), anterolateral view (B) and medial surface of the prootic (C).

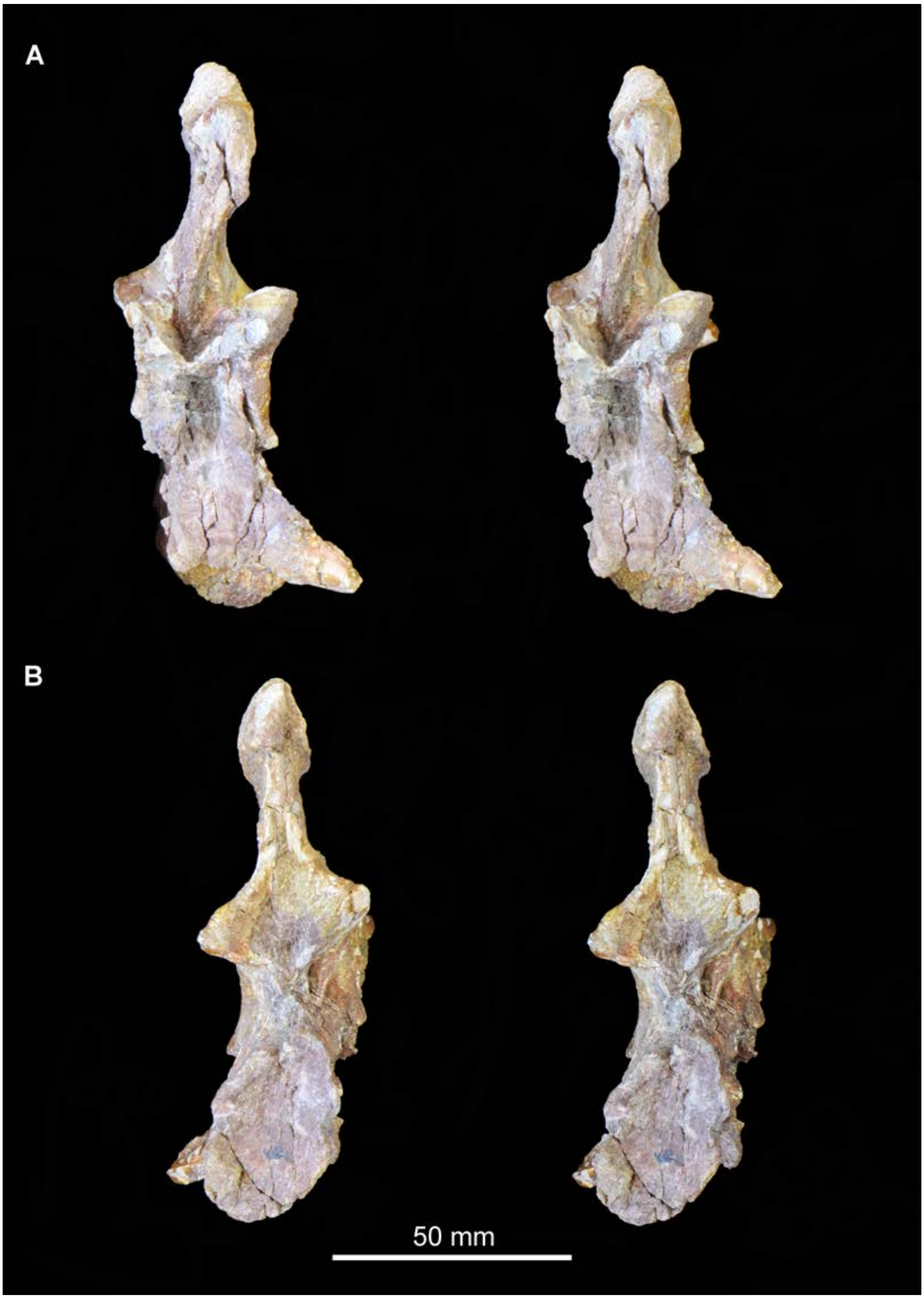

Figure S10. ‘Cervical a’ of *Guchengosuchus shiguaiensis*, IVPP V8808, stereopairs in anterior (A) and posterior (B) views.

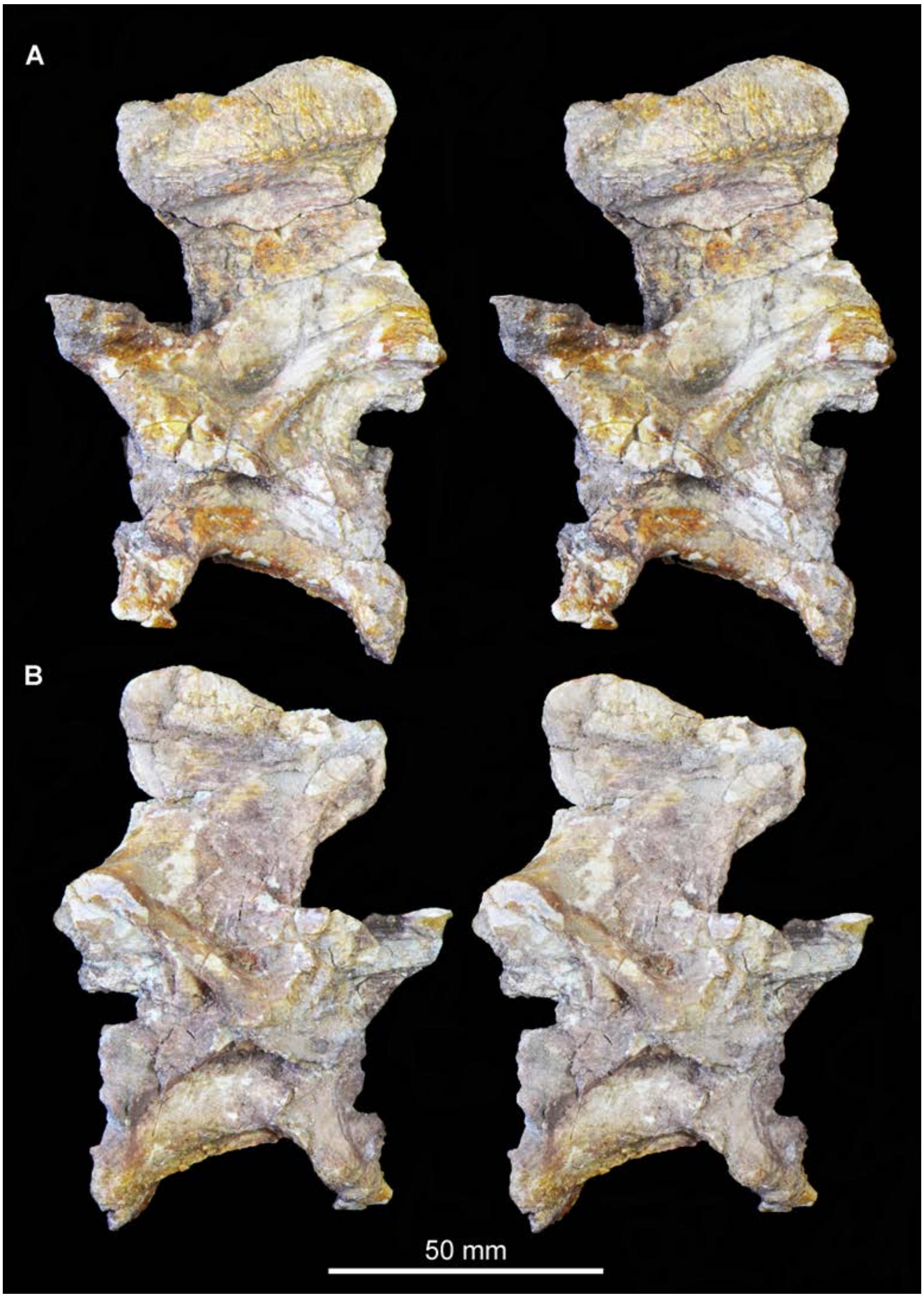

Figure S11. 'Cervical a' of *Guchengosuchus shiguaiensis*, IVPP V8808, stereopairs in left lateral (A) and right lateral (B) views.

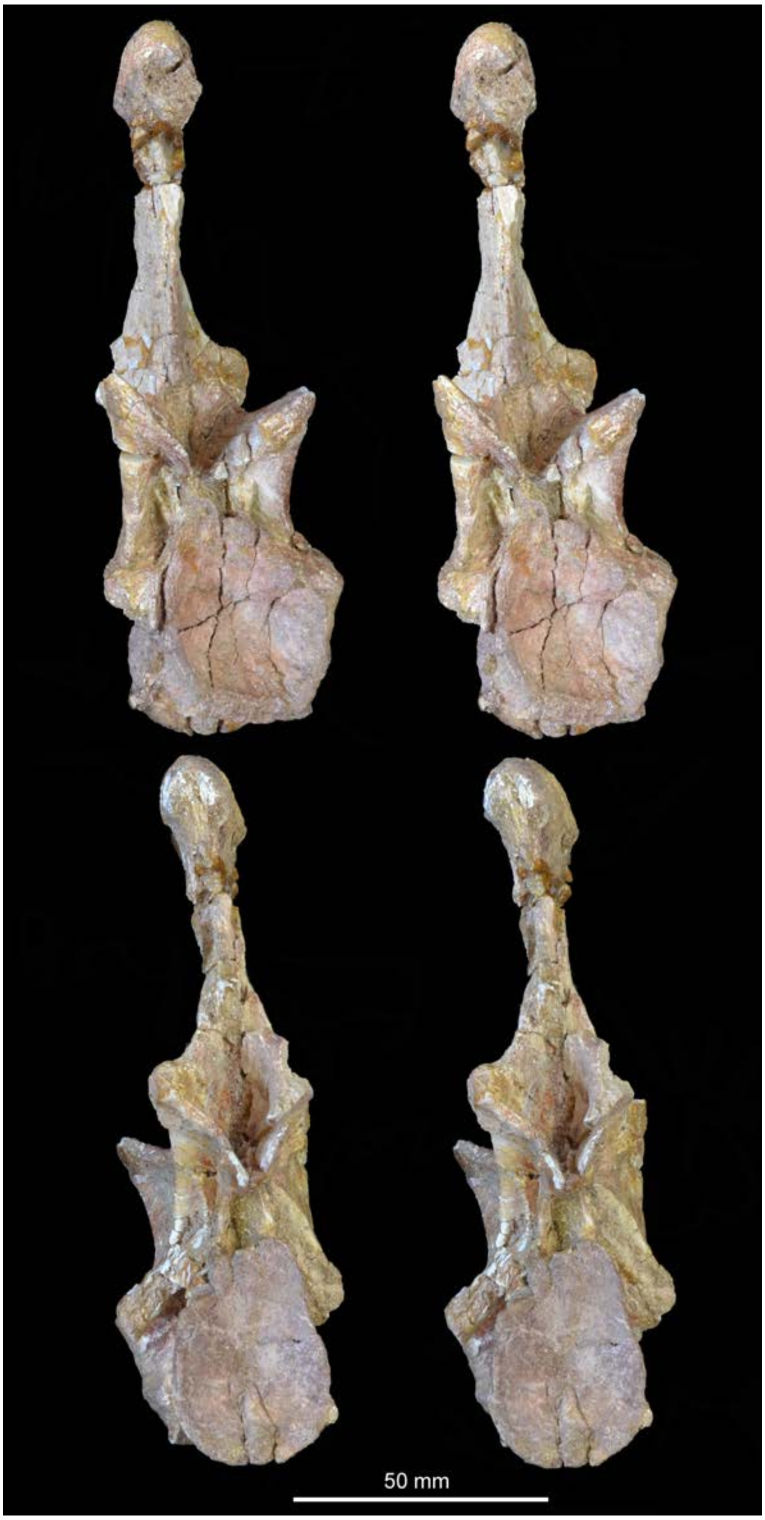

Figure S12. ‘Cervical b’ of *Guchengosuchus shiguaiensis*, IVPP V8808, stereopairs in anterior (A) and posterior (B) views.

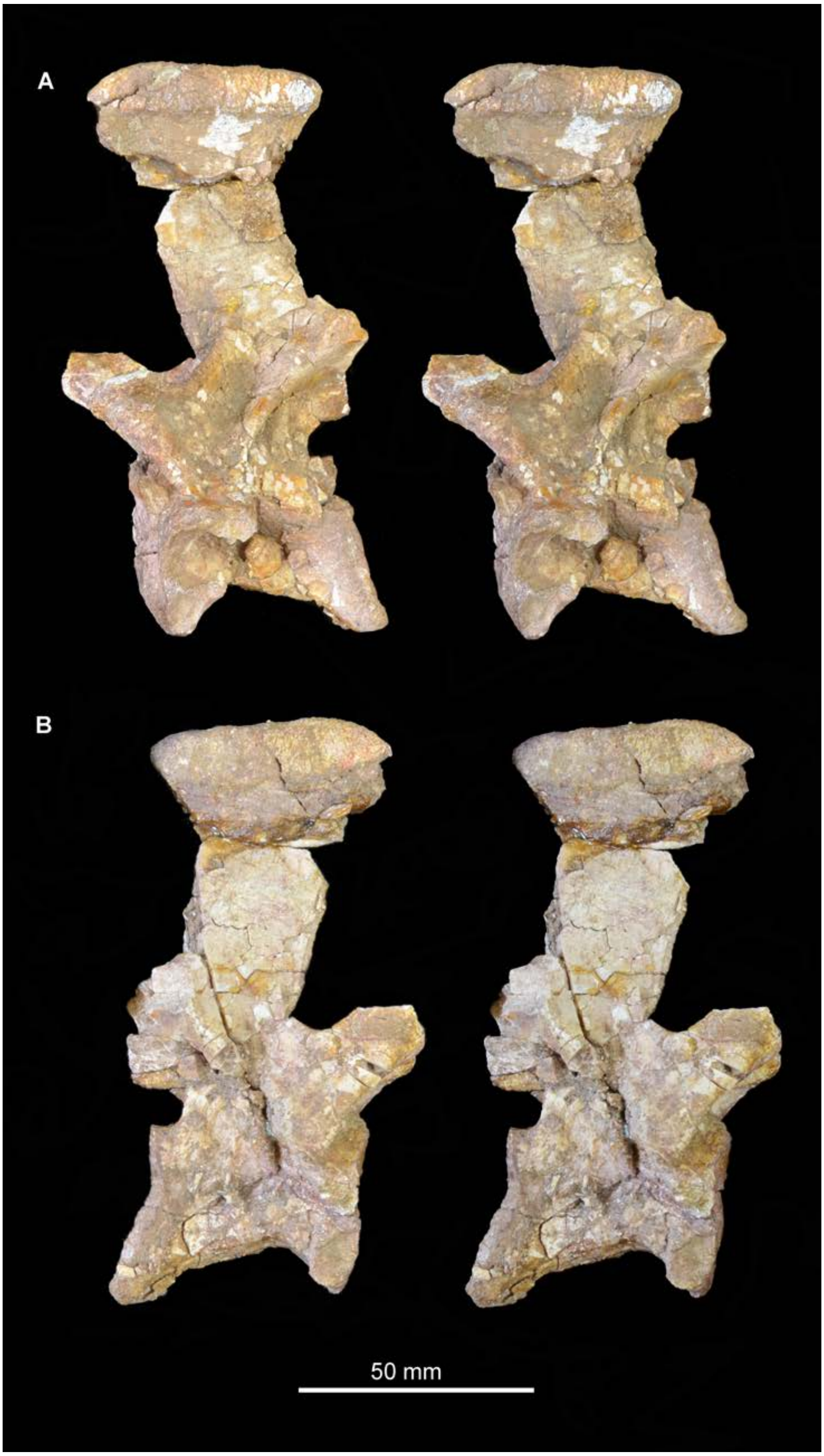

Figure S13. ‘Cervical b’ of *Guchengosuchus shiguaiensis*, IVPP V8808, stereopairs in left lateral (A) and right lateral (B) views.

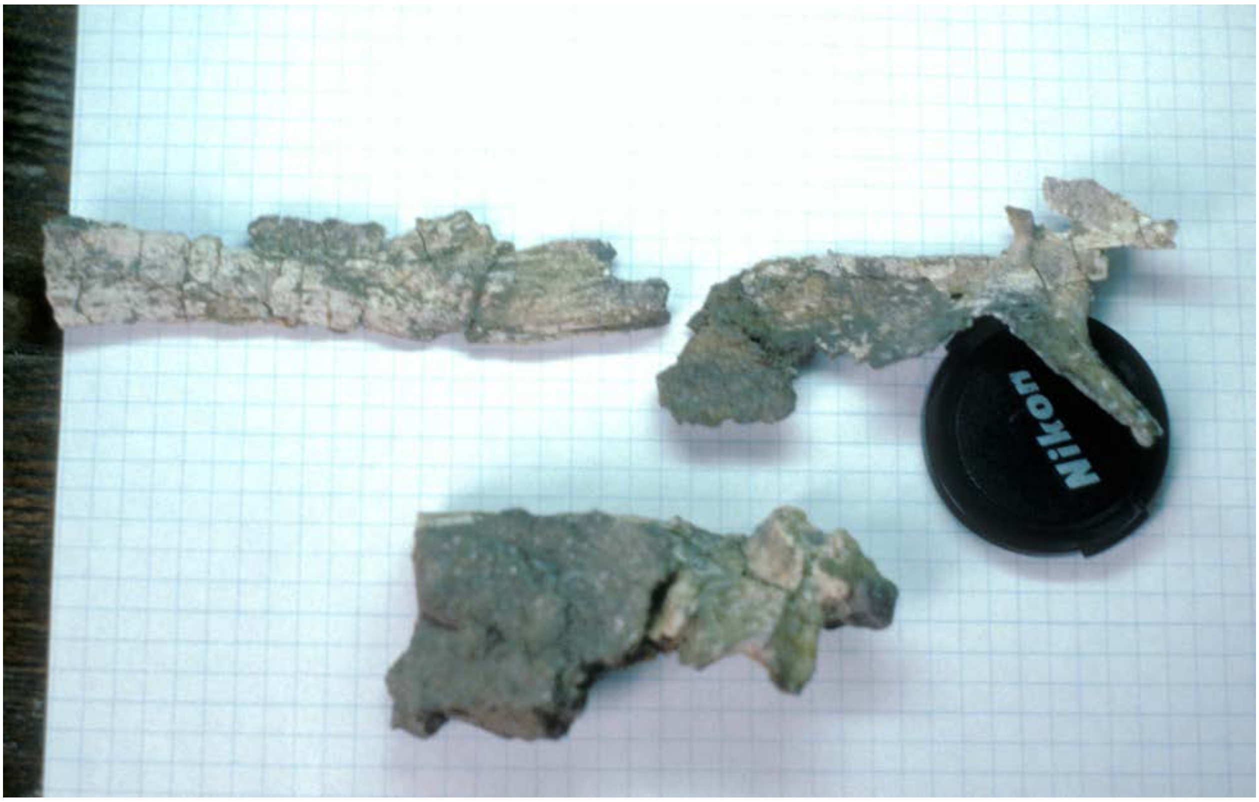

Figure S14. Cranial remains of *Guchengosuchus shiguaiensis*, IVPP V8808, as photographed by Michael Parrish in 1990. Image shows left nasal (top left), left pterygoid (top right), and posterior right mandible (bottom). Lens cap is 52 mm in diameter.

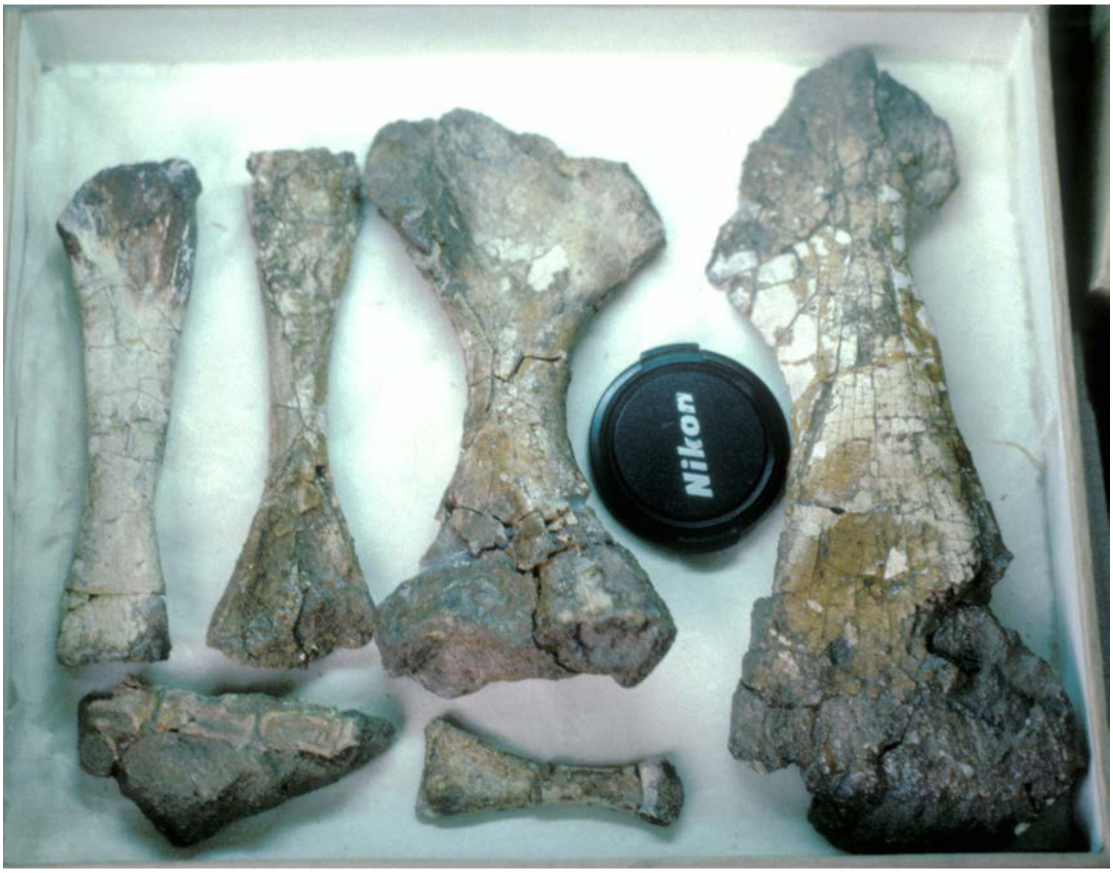

Figure S15. Postcranial remains of *Guchengosuchus shiguaiensis*, IVPP V8808, as photographed by Michael Parrish in 1990. At the top, from left to right, are the right ulna, right radius, right humerus (anterior view), and right scapula (lateral view0. At the bottom are what appear to be phalanges (left) and a metapodial (right). Lens cap is 52 mm in diameter.

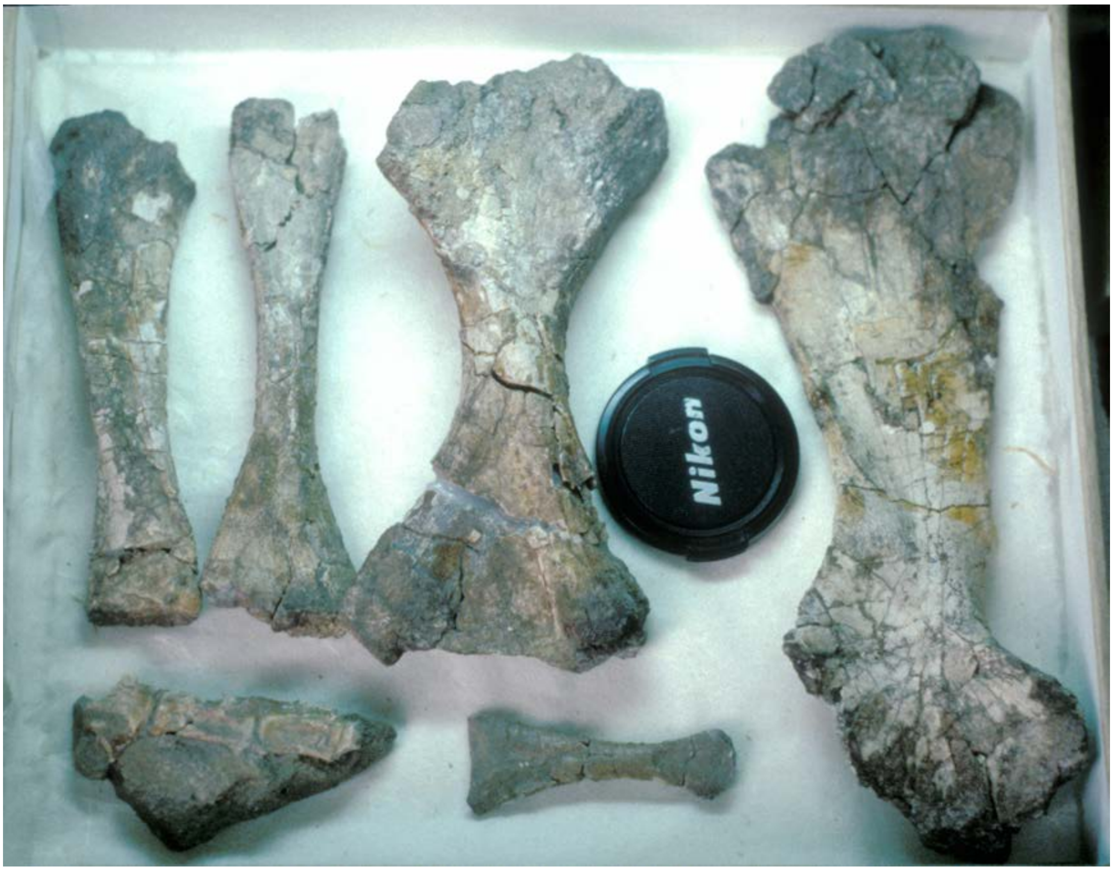

Figure S16. Postcranial remains of *Guchengosuchus shiguaiensis*, IVPP V8808, as photographed by Michael Parrish in 1990. At the top, from left to right, are the right ulna, right radius, right humerus (posterior view), and right scapula (medial view). At the bottom are what appear to be phalanges (left) and a metapodial (right). Lens cap is 52 mm in diameter.
